# Phytoplankton seasonal succession in response to environmental drivers in the eastern English Channel: a revised Margalef Mandala for a highly turbulent region

**DOI:** 10.1093/plankt/fbag086

**Published:** 2026-11-12

**Authors:** Raed Halawi Ghosn, Kelly Grassi, Emilie Poisson-Caillault, Alain Lefebvre

**Affiliations:** Ifremer, Unité COAST, Laboratoire Environnement et Ressources, 62200 Boulogne-sur-Mer, France; Ifremer, Unité COAST, Laboratoire Environnement et Ressources, 62200 Boulogne-sur-Mer, France; LISIC, UR 4491 ULCO-Université du Littoral Côte d’Opale, 62228 Calais, France; LISIC, UR 4491 ULCO-Université du Littoral Côte d’Opale, 62228 Calais, France; Ifremer, Unité COAST, Laboratoire Environnement et Ressources, 62200 Boulogne-sur-Mer, France

**Keywords:** Margalef Mandala, phytoplankton dynamics, *Phaeocystis globosa*, multilevel spectral clustering (M-SC), eastern English Channel

## Abstract

Classical conceptual models or Mandalas may explain phytoplankton dynamics in ecosystems where succession from diatoms to dinoflagellates is commonly observed. In highly turbulent, diatom-*Phaeocystis* dominated waters of the eastern English Channel, however, these models may not be fully applicable. This study identifies environmental states and key drivers of phytoplankton seasonal succession in the eastern English Channel by integrating high-frequency hydrological and physicochemical data with low-frequency phytoplankton observations. Multilevel Spectral Clustering (M-SC) was applied to a high-frequency dataset to identify environmental states, which were then linked to a phytoplankton abundance dataset. Eight environmental states were identified, including recurrent, rare, and extreme events, revealing a succession from winter conditions dominated by tychopelagic and other diatoms (e.g. *Skeletonema costatum*, *Thalassiosira*, *Thalassionema*), to spring blooms driven by *Phaeocystis globosa* and *Pseudo-nitzschia*. A transition period precedes summer assemblages dominated by diatoms (e.g. *Rhizosolenia*, *Chaetoceros*, *Guinardia*, *Leptocylindrus)*. Diatoms persist year-round with spring blooms of *P. globosa*, highlighting their ecological plasticity, while dinoflagellates remain scarce. Inspired by Margalef Mandala, a Margalef-based visualization diagram was developed. The revised Mandala maps phytoplankton across six environmental variables including nitrate and nitrite, phosphate, silicate, photosynthetically active radiation, turbidity, and nitrate-to-phosphate ratio. This study may provide new insights to predict phytoplankton assemblages in the context of climate change and anthropogenic pressure.

## INTRODUCTION

Understanding phytoplankton seasonal succession and composition is a central focus in marine ecology due to their pivotal role in primary production, ocean biogeochemical cycles, trophic energy transfer, the biological carbon pump, and climate regulation (Falkowski *et al.,* 2003; Falkowski, 2012; Not *et al.,* 2012; Behrenfeld *et al.,* 2021). Phytoplankton succession and dynamics are controlled by several abiotic factors including light, nutrients, temperature and turbulence, as well as biotic factors such as grazing and allelopathy (Reynolds, 2006; Litchman and Klausmeier, 2008; Allen and Polimene, 2011). There is thus a keen interest in modeling phytoplankton succession across environmental gradients.

A pioneer in plankton ecology, Ramon Margalef, developed a series of conceptualization models, termed Mandalas, that map phytoplankton across two gradients: nutrients and turbulence (Margalef, 1958, 1967, 1978). In his Mandalas, diatoms thrive in turbulent, nutrient rich waters, while dinoflagellates predominate in stratified, nutrient poor waters (Margalef, 1977, 1978). There have been recent attempts to update the original Mandala (Cullen *et al.,* 2002, 2007; Kudela, 2010; Allen and Polimene, 2011; Glibert, 2016; Behrenfeld *et al.,* 2021), as the original was limited by sampling strategies and knowledge of its era, and dealt mainly with two environmental variables.

In the macrotidal, highly turbulent waters of the eastern English Channel, where little to no stratification is observed (Brylinski *et al.,* 1991), original and modified versions of Mandalas may not be fully applicable to describe phytoplankton succession. In this well-mixed temperate ecosystem, decades of research have shown the dominance of diatoms and the spring blooms of the Prymnesiophyceae *P. globosa* (Schapira *et al.,* 2008; Lefebvre *et al.,* 2011; Bonato *et al.,* 2016; Breton *et al.,* 2021; Skouroliakou *et al.,* 2024), where dinoflagellates are only present at relatively low abundance. There is therefore a need to further recognize the diverse ecology of diatoms, as proposed by Kemp and Villareal (2018), as well as cryptophytes, dinoflagellates, and algal blooms of the Prymnesiophyceae *P. globosa,* and to propose a revised Mandala.

Despite the advances in oceanographic measurements (Dickey and Bidigare, 2005; Chai *et al.,* 2020; Marcelli *et al.,* 2026), and phytoplankton monitoring programs, there remains a need to improve our understanding of the links between environmental variables, phytoplankton succession, and environmental states. The main limitation lies in the reliance on traditional statistical approaches that are not well-suited for analyzing non-linear, multivariate time series. In this context, Grassi *et al.* (2019) developed multi-level spectral clustering (M-SC) to segment marine time series without any *a priori* knowledge. The importance of this methodology lies in its ability to extract general patterns from time series, which can help us understand ecosystem dynamics by detecting general, recurrent, extreme, as well as rare events, and then link each event to its corresponding physicochemical variables (Grassi *et al.,* 2020).

The aim of this research is to (1-) determine the different coastal environmental states in the eastern English Channel using unsupervised clustering, (2-) identify the phytoplankton community composition of each state by integrating data from different sources, and (3-) develop a Margalef-based visualization diagram that plots phytoplankton succession across the main variables controlling phytoplankton dynamics in this region.

## METHODS

### High-frequency MAREL Carnot dataset

The high frequency dataset used in this research is derived from an automated oceanographic station installed in the eastern English Channel, called MAREL Carnot. This station belongs to a network of fixed stations along the French coast (https://coast-hf.fr; last access: 08 April 2026), which is part of the research infrastructure ILICO (www.ir-ilico.fr, last access: 08 April 2026). The station provides in-situ measurements of physicochemical variables every 20 minutes, except for nutrients (nitrate, phosphate and silicate) which are measured every 12 hours (Halawi Ghosn *et al.,* 2023). The MAREL Carnot dataset is freely accessible (https://doi.org/10.17882/39754;  MAREL Carnot, 2023) and processed via offset correction, quality control, sensor and expert range correction, as well as time alignment. For further details about the data preprocessing steps, see Halawi Ghosn *et al.* (2023).

For this research, data from 2005 to 2009 included were used since these years contain data on nutrients such as phosphate, silicate and nitrate. Nine variables were selected to provide a comprehensive representation of the physical, chemical, and biological conditions of the study area, which are known to control phytoplankton dynamics and allow the identification and characterization of distinct environmental states. These variables include water temperature (°C), salinity (PSU), turbidity (NTU), dissolved oxygen (mg.L^−1^), fluorescence (FFU), photosynthetically active radiation (PAR) (μE.m^−2^.s^−1^), phosphate (μmol.L^−1^), silicate (μmol.L^−1^), and nitrate+nitrite (μmol.L^−1^). Together, these variables capture key environmental drivers including hydrography, light availability, oxygen conditions, and nutrient concentrations thus providing a reduced yet comprehensive set of descriptors commonly used in European directives and regional sea conventions for environmental assessment. It is important to highlight that PAR is not representative of the actual light available for photosynthesis because the PAR sensor is placed at 28 meters above sea surface, i.e. the light attenuation in the water due to particles is not integrated in this research.

To account for missing values (NA; *Not Available*), data completion was performed using an improved version of the Dynamic Time Warping Based Imputation (DTWBI) method (Phan *et al.,* 2017). Isolated holes (single holes) were completed with the local average. Two parameters (small-size hole and accepted-size hole) were tuned to adapt the size of the NA completion windows (Table I). The small-size holes and accepted size holes were completed by weighted moving average and DTWBI, respectively. Based on tuning, 3 days and 1 month were selected to avoid a plateau effect and prevent the completion of long periods (Table I). The completed data from 01-01-2005 to 31-12-2009 contains 131 472 observations (N) and 9 variables.

**Table I:** Percentage of available data (%) before and after data completion with the size of small and accepted holes (*: Selected small and accepted hole)

Small-size Hole	Accepted-size Hole	Percentage of Available Data (%)
-	-	0
1 day	7 days	47.62
1 day	15 days	50.09
1 day	21 days	50.47
1 day	1 month	50.47
1 day	2 months	50.95
3 days	1 month	59.34*
1 Week	1 month	62.82

### Multilevel spectral clustering (M-SC)

Following data completion, Multilevel Spectral Clustering (M-SC) was performed to identify contrasted environmental states. M-SC, introduced by Grassi *et al.* (2019), combines spectral clustering, similar to NJW-Spectral Clustering (Ng *et al.,* 2001), with a deep hierarchical architecture. This permits event identification ranging from global in the upper levels to specific or detailed in the lower levels. Thus, when applied to the MAREL Carnot dataset, it enables the definition of environmental states associated with phytoplankton dynamics, i.e. recurrent, extreme and rare events. To clarify, each cluster with a given structure (combination of data) in the spectral space represents an environmental state such as a bloom or an extreme event.

Multilevel Spectral Clustering was performed as per Grassi *et al.* (2020). Accordingly, Spectral Partition around Medoids (Spectral-PAM) was recursively applied to data normalized in clusters. The number of clusters was automatically identified from the principal eigenvalue method (PEV), by counting dominant eigenvalues greater than a threshold of 0.9. If the number of clusters could not be identified, then the dataset would be bipartitioned as per Shi and Malik (1997). Moreover, a silhouette of 0.7 was used to limit over-segmentation while detecting short-duration events (Grassi *et al.,* 2020). Lastly, the maximum levels of clustering considered for this research was three, consistent with the ecological interpretability required for this study.

The dataset was thus divided into two, four and eight clusters at levels one, two and three, respectively. A cluster labelled NA (Not Available) was added to represent unclassified observations resulting from at least one unavailable variable for a given date. Then, a label was assigned to each cluster. It should be mentioned that generic names were used for simplicity. For instance, a cluster that persists throughout the year, but predominantly during winter was labelled as winter period.

### Low-frequency REPHY-SRN dataset

Phytoplankton abundance and taxonomic composition in surface waters (0–1 meters) along the entire French metropolitan coast were provided by the REPHY (“REseau d’Observation et de Surveillance du Phytoplancton et de l’Hydrologie dans les eaux littorales”) and SRN (“Suivi Régional des Nutriments”) networks. These networks are part of the National Observation Services SNO PHYTOBS Network which coordinates phytoplankton observation at theFrench national scale (PHYTOBS, 2023), which in turn is part of the research infrastructure ILICO.

Phytoplankton samples were collected at low temporal resolution, ranging from fortnightly to monthly (Belin *et al.,* 2021; Lefebvre and Devreker, 2023). These samples were preserved in Lugol solution, then settled in a counting chamber according to Utermöhl method (Utermöhl, 1958). Cells were counted using inverted microscopy. Included in the count were phytoplankton measuring more than 20 μm and smaller species forming chains or colonies or considered potentially harmful (example: *P. globosa*). For further details on counting, see Lefebvre *et al.* (2011), Belin and Neaud-Masson (2012), and Neaud-Masson (2015).

The REPHY-SRN dataset was downloaded from the Quadrige2 website (https://quadrige.ifremer.fr/, last access: 23 April 2024) and taxa were grouped into Operational Taxonomic Units (OTUs). Such grouping helps overcome identification difficulties between species or genera using optical microscopy (Hernández Fariñas *et al.,* 2015; Karasiewicz *et al.,* 2020). This was the case for *Pseudo-nitzschia spp.* where thin cells are grouped together as *Pseudo-nitzschia delicatissima* complex, and large or wide cells are grouped together as *Pseudo-nitzschia seriata* complex. The use of operational taxonomic units guarantees taxonomic heterogeneity over time.

Following this grouping, the total flora data from the Boulogne-sur-Mer radial station (BL1) located near the MAREL Carnot station, were selected for the 2005–2009 period. To account for differences in temporal resolution of the datasets, the REPHY-SRN dataset was labelled by selecting the most frequent label within a one week window (3 days before and after the specific date). Finally, dominant taxa for each cluster were identified as those contributing a cumulative abundance of up to 95% of the total.

### Margalef-based visualization diagram

To provide a comprehensive overview of the characteristics of each environmental state (cluster), we propose an interpretation diagram derived from Margalef’s species succession model (Margalef, 1978). The proposed diagram represents a compromise between the original Mandala, which considers only two variables (Nutrients and Turbulence), and a complex modeling approach incorporating all the mechanisms and processes which results in too many variables for an expert to handle during interpretation.

The proposed Margalef-based visualization diagram takes into account the balance between nutrient availability, light conditions and mixing degrees, based on 6 explanatory variables: Turbidity (Turb), Photosynthetically active radiation (PAR), Nitrate + Nitrite (N), Phosphate (P), Silicate (S), and calculated Nitrate-to-Phosphate Ratio (NP). Fluorescence was excluded because it represents a proxy of phytoplankton biomass (i.e. a response variable) rather than an independent environmental driver. Dissolved oxygen was also excluded because it reflects ecosystem state (i.e. a response variable) rather than an environmental gradient. Salinity was not retained because it did not provide additional explanatory information. This reduced set of variables allows a minimal yet comprehensive representation of the key environmental gradients structuring phytoplankton communities. This diagram thus links the environmental states to their corresponding environmental variables, and to phytoplankton taxa.

To create the diagram, cluster barycenters were calculated and normalized (center-reduced) using minimum-maximum scaling. The variables were then represented pairwise in an ensemble graph where each cluster is designated by its number and color. This diagram combines the six variables, two by two in a matrix of plots manner using R software (R Core Team, 2022), thus allowing multivariate information to be summarized while retaining the possibility of analyzing each variable individually.

A revised phytoplankton Mandala is then proposed that is suitable for the highly turbulent eastern English Channel. It is a revision of the Mandala presented by Glibert (2016). In order to assess the applicability of the revised Mandala under current environmental conditions, it was compared with a recent dataset from 2021 from the REPHY-SRN monitoring program. This comparison was intended to evaluate whether the main phytoplankton succession patterns described by the revised Mandala remain consistent despite potential interannual and climate-driven environmental variability in the eastern English Channel.

## RESULTS

### Environmental states obtained at level 1 clustering (M-SC at level 1)

The environmental states identified at level 1 clustering primarily reflected differences in phytoplankton biomass. Two contrasting environmental states (clusters) were identified, corresponding to high- and low- biomass periods (Fig. 1a). The high biomass period (Cluster 2) was characterized by higher turbidity (max. = 237.4 NTU) and fluorescence (max. = 58.31 FFU), as well as elevated nutrient concentrations, with nitrate + nitrite, phosphate and silicate reaching maxima of 98.89, 10 and 34.64 μmol L^−1^, respectively (Fig. 2). In contrast, the low biomass period (Cluster 1) exhibited lower turbidity (max. = 70.5 NTU) and fluorescence (max. = 14.07 FFU), but higher temperature (median = 16.2°C), PAR and salinity (Fig. 2).

**Fig. 1:**
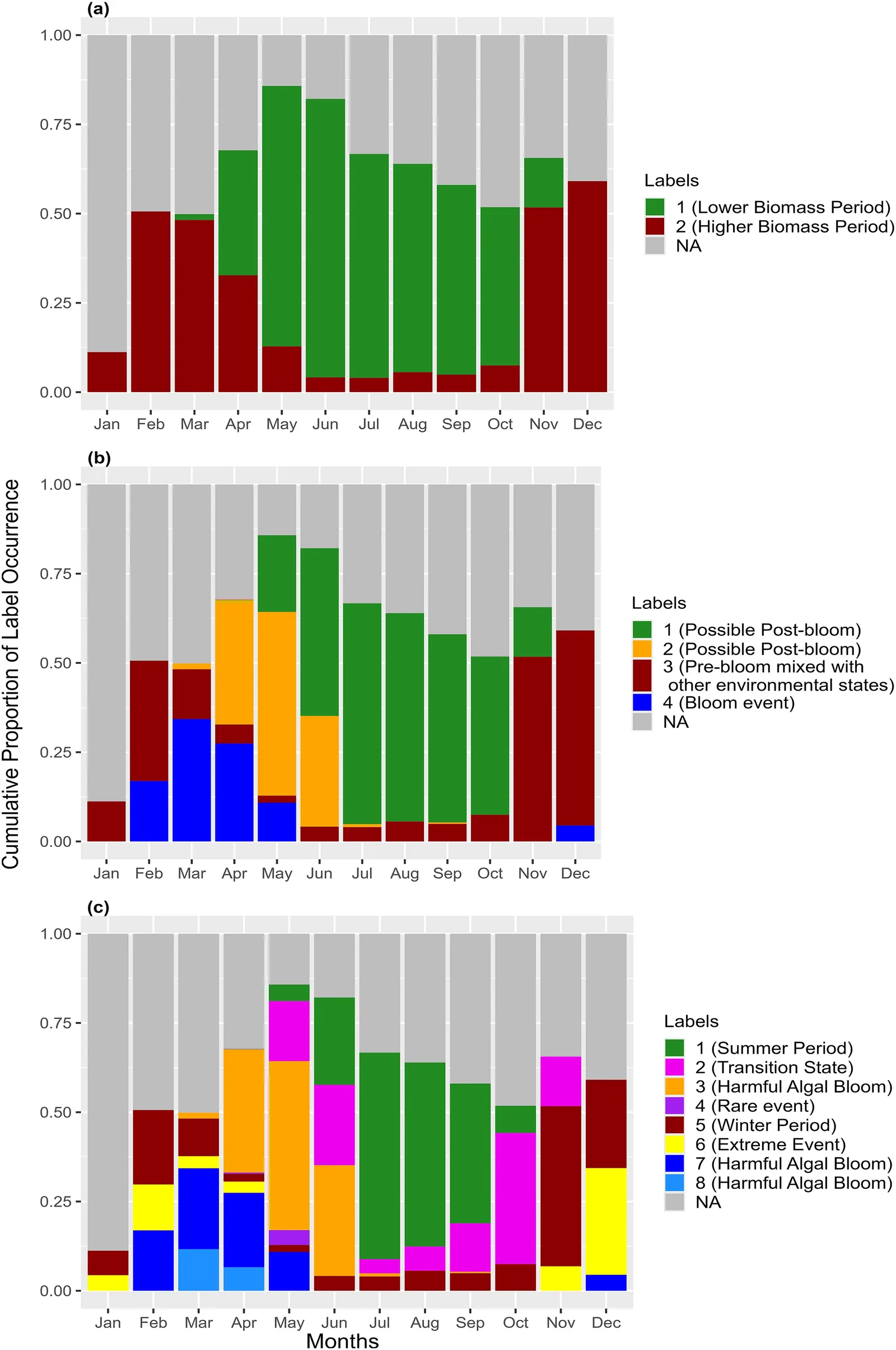
A summary representing the mean dynamics of each environmental state by month over the period 2005–2009, showing the cumulative label occurrence by month at (a) Level 1, (b) Level 2, and (c) Level 3 clustering. The labels presented on the right were determined based on environmental variables and phytoplankton data. (NA, Not Available, represents the part of unclassified observations due to at least one unavailable variable for a given date).

**Fig. 2:**
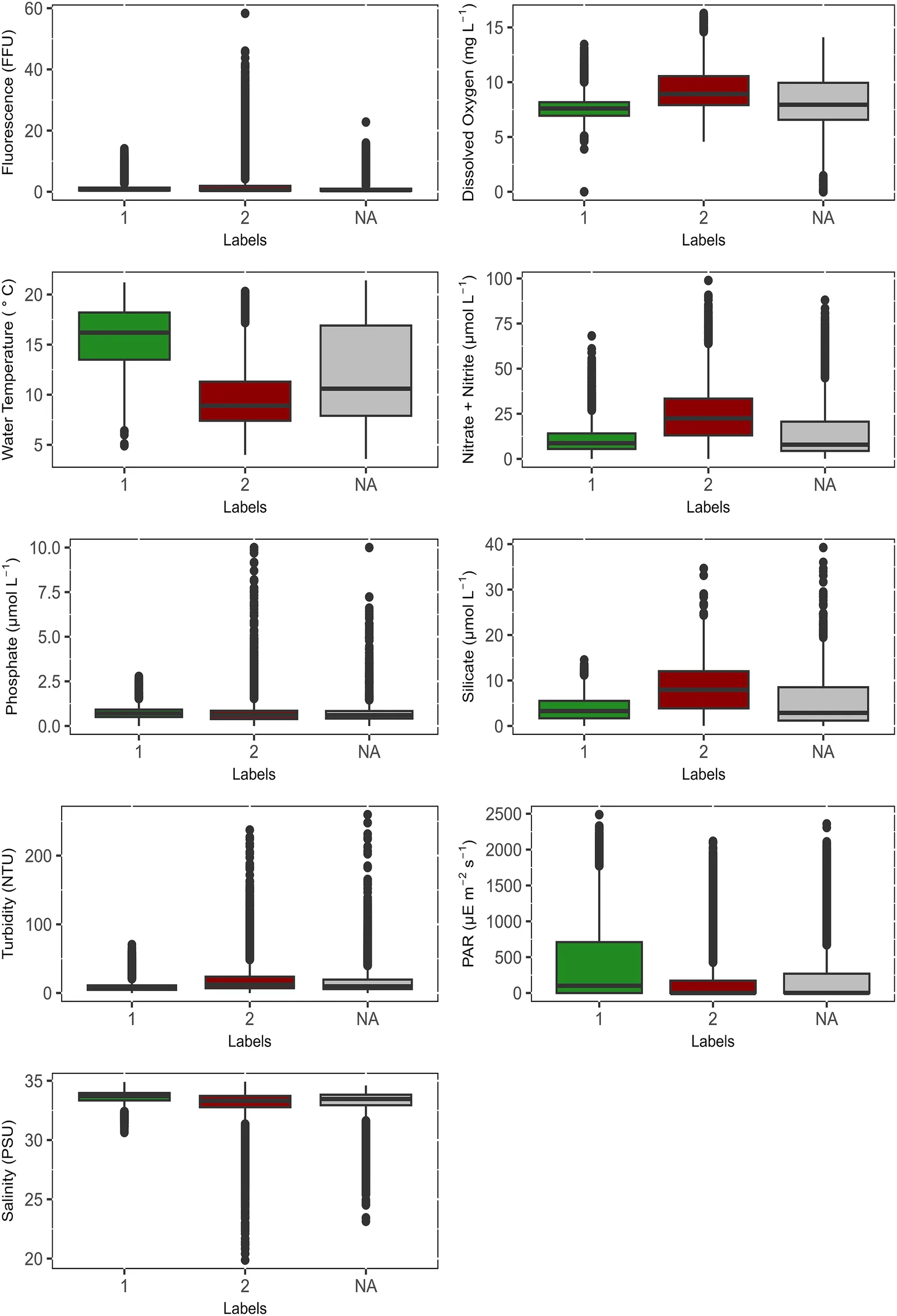
The main statistical characteristics of the nine variables at level 1 clustering. The boxes represent the interquartile range (IQR), from the 25th to the 75th percentile, and the horizontal line within each box indicates the median. The vertical whiskers indicate the range of observations, while the dots represent observations beyond the whiskers (outliers).

### Environmental states obtained at level 2 clustering (M-SC at level 2)

Four environmental states (clusters), exhibiting partial overlap and limited separation, were identified at level 2 clustering and corresponded to pre-bloom, bloom and post-bloom (Fig. 1b). The pre-bloom (Cluster 3) was characterized by intermediate fluorescence (median = 0.44 FFU), high nutrient concentrations, including nitrate + nitrite (max. = 98.89 μmol L^−1^), phosphate (max. = 10 μmol L^−1^), and silicate (median = 9.47 μmol.L^−1^), with high turbidity (median = 8.5 NTU) and low salinity levels (min. = 20 PSU) (Fig. 3). The bloom state (Cluster 4) subsequently exhibited the highest fluorescence (median = 3.83 FFU), and dissolved oxygen (median = 10.9 mg.L^−1^), while nutrient concentrations were lower than those observed in the pre-bloom state (Cluster 3) (Fig. 3). Two possible post-bloom states (clusters 1 and 2) were then identified, differing primarly in PAR and water temperature. Cluster 2 had higher PAR (median = 274.5 μE.m^−2^.s^−1^) than Cluster 1 (median = 60.7 μE.m^−2^.s^−1^), whereas cluster 1 exhibited higher water temperature (median = 17.4°C) than Cluster 2 (median = 12.1°C) (Fig. 3).

**Fig. 3:**
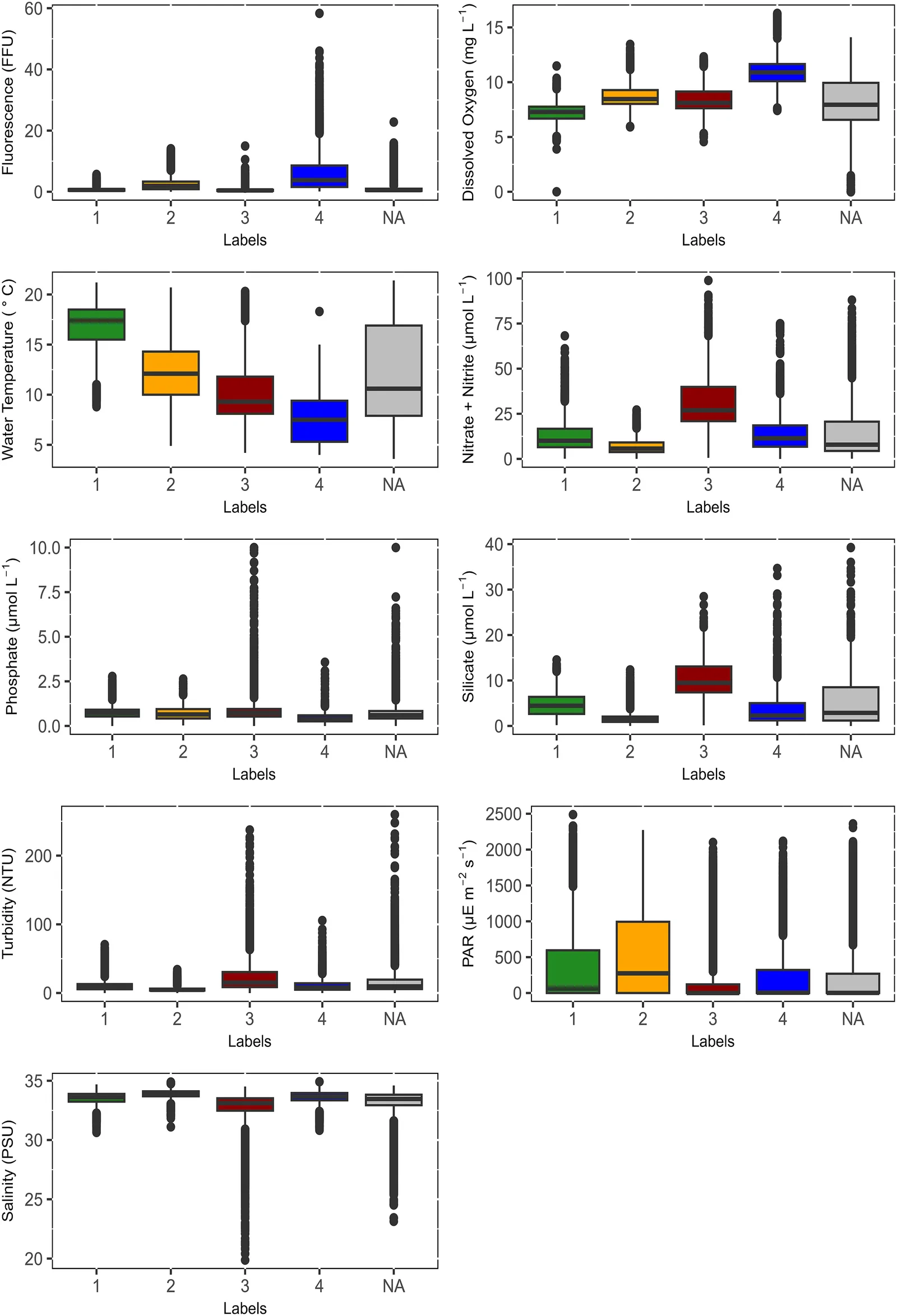
The main statistical characteristics of the nine variables at level 2 clustering. The boxes represent the interquartile range (IQR), from the 25th to the 75th percentile, and the horizontal line within each box indicates the median. The vertical whiskers indicate the range of observations, while the dots represent observations beyond the whiskers (outliers).

### Environmental states at level 3 clustering and phytoplankton assemblage (M-SC at level 3, and REPHY-SRN phytoplankton taxa)

Eight environmental states (clusters) were identified at level 3 clustering, capturing seasonal succession patterns as well as rare and extreme events (Fig. 1c). These states are described below.

#### Winter period

The winter period (Cluster 5) was characterized by low light levels (with an approximate PAR median and mean of 0.6 and 150 μE.m^−2^.s^−1^, respectively) and high concentration of nutrients (approximate mean of nitrate +nitrite, phosphate and silicate were 25, 1, and 10 μmol L^−1^, respectively). This period was also characterized by low levels of salinity (approximate mean of 33 PSU and a minimum of 20 PSU), and a low phytoplankton biomass, with a fluorescence median of 0.44 FFU and mean 0.6 FFU (Fig. 4).

**Fig. 4:**
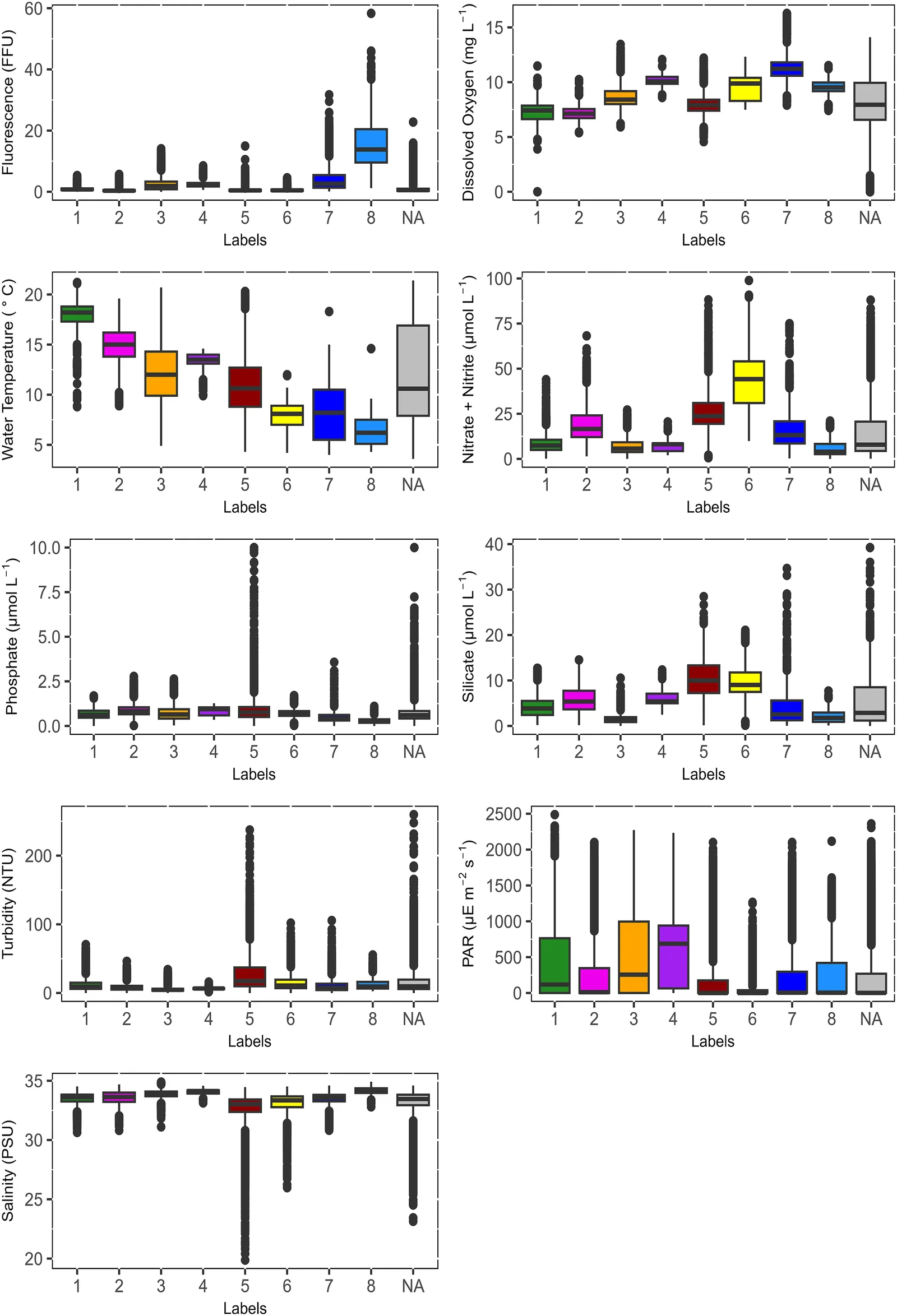
The main statistical characteristics of the nine variables at level 3 clustering. The boxes represent the interquartile range (IQR), from the 25th to the 75th percentile, and the horizontal line within each box indicates the median. The vertical whiskers indicate the range of observations, while the dots represent observations beyond the whiskers (outliers).

The phytoplankton taxonomic assemblage in winter included *Skeletonema costatum* (25.7%), *Cryptophyceae* (24.9%), *Paralia sulcata* (9.4%), *Rhaphoneis—Delphineis* (6.8%), *Chaetoceros* (6.7%), *Thalassiosira* (4.1%), *Gymnodiniales* (2.9%), and *Thalassionema nitzschioides* (1.1%) (Fig. 5).

**Fig. 5:**
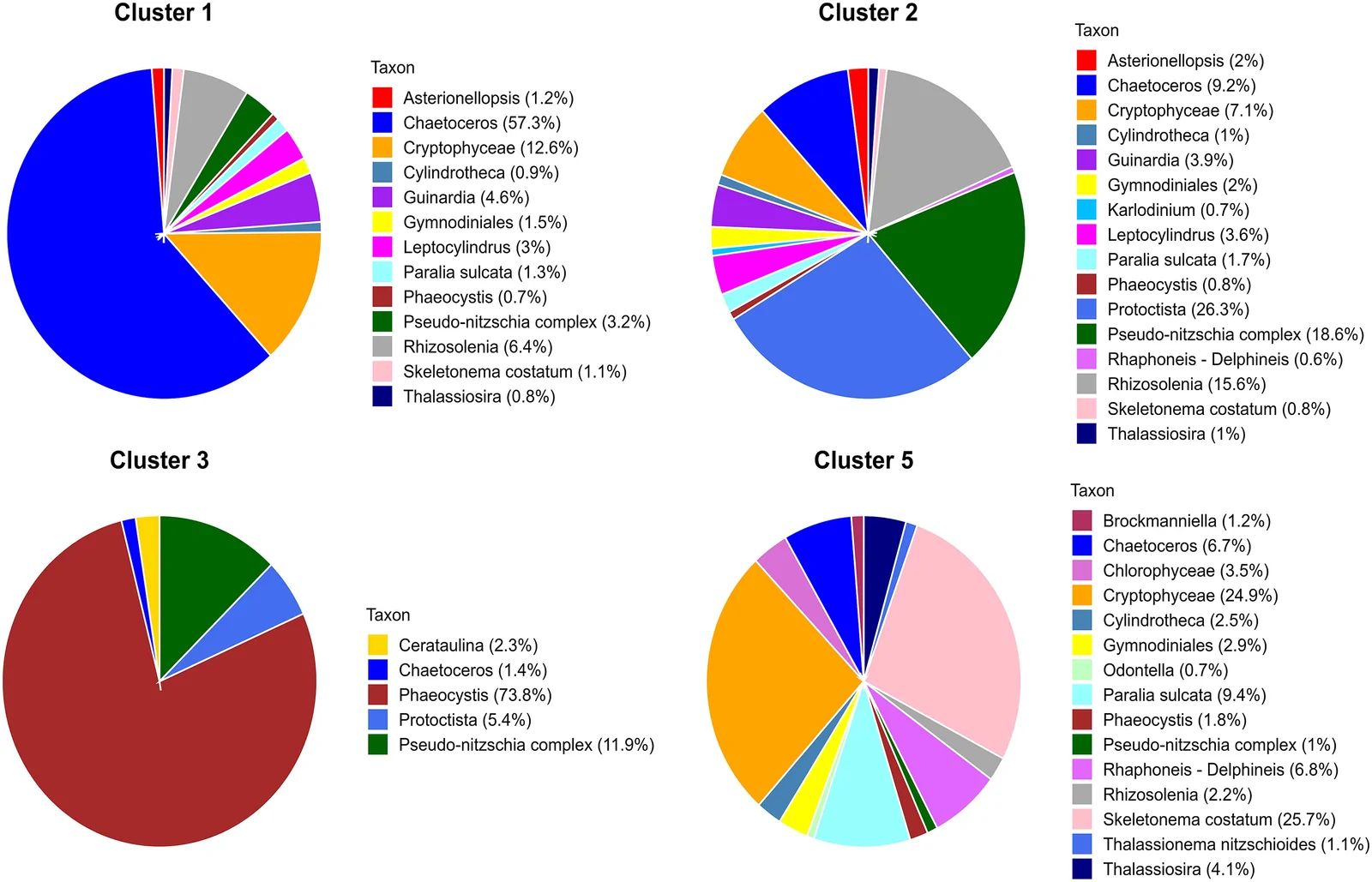
Relative abundance of dominant phytoplankton taxa (OTU) in clusters 1, 2, 3 and 5. The cumulative abundance of the taxa represented in each of these clusters accounts for > 94% of the total abundance.

#### Spring blooms

Three algal bloom events were identified for the spring bloom, revealing two successive phases and a short-lived bloom occurring intermittently in 2005, 2006 and 2008 (Clusters 7, 3 and 8) (Fig. 1c). To clarify, the spring bloom was divided into two phases, an early phase (Cluster 7) followed by a second phase of the bloom (Cluster 3), whereas Cluster 8 represented a short-lived bloom state that occurred only during these three years. All these bloom events (clusters) exhibited high fluorescence, reaching a maximum of 31.71 FFU in Cluster 7 and 58.31 FFU in Cluster 8 which decreased to 14.07 FFU during the second phase of the bloom (Cluster 3) (Fig. 4).

The first phase of the bloom (Cluster 7) was characterized by high nitrate + nitrite concentrations, with a median of 13.12 μmol L^−1^ and maximum 74.93 μmol L^−1^. Phosphate and silicate were lower than in winter (Cluster 5), with a median of 0.4 and 2.52 μmol L^−1^, respectively. PAR was intermediate with a mean of 226.76 μE m^−2^ s^−1^, while dissolved oxygen reached its highest values, with a maximum of 16.28 mg L^−1^ (Fig. 4).

As the spring bloom progressed into the second phase (Cluster 3), nitrate + nitrite further decreased to a median of 5.69 μmol L^−1^. Similarly, silicate decreased to a median of 1.34 μmol L^−1^, while phosphate increased slightly to 0.64 μmol L^−1^. PAR further increased in this stage reaching a mean of 528.76 μE m^−2^ s^−1^ (Fig. 4).

The bloom events also differed in their phytoplankton composition. *P. globosa* was present along with *Chaetoceros* (2.5%) during the first phase of the bloom (Cluster 7). During the second phase (Cluster 3) *P. globosa* was dominant (73.8%) but co-occurred with other taxa including *Pseudo-nitzschia* complex (11.9%), *Protoctista* (5.4%), *Cerataulina* (2.3%), and *Chaetoceros* (1.4%). In contrast, *P. globosa* was the only phytoplankton taxon identified in Cluster 8 (Fig. 5 and Fig. 6). Overall, clusters 7 and 8 correspond to *P. globosa*-dominated harmful algal blooms, whereas Cluster 3 additionally included potentially toxic taxa, notably *Pseudo-nitzschia spp.*

**Fig. 6:**
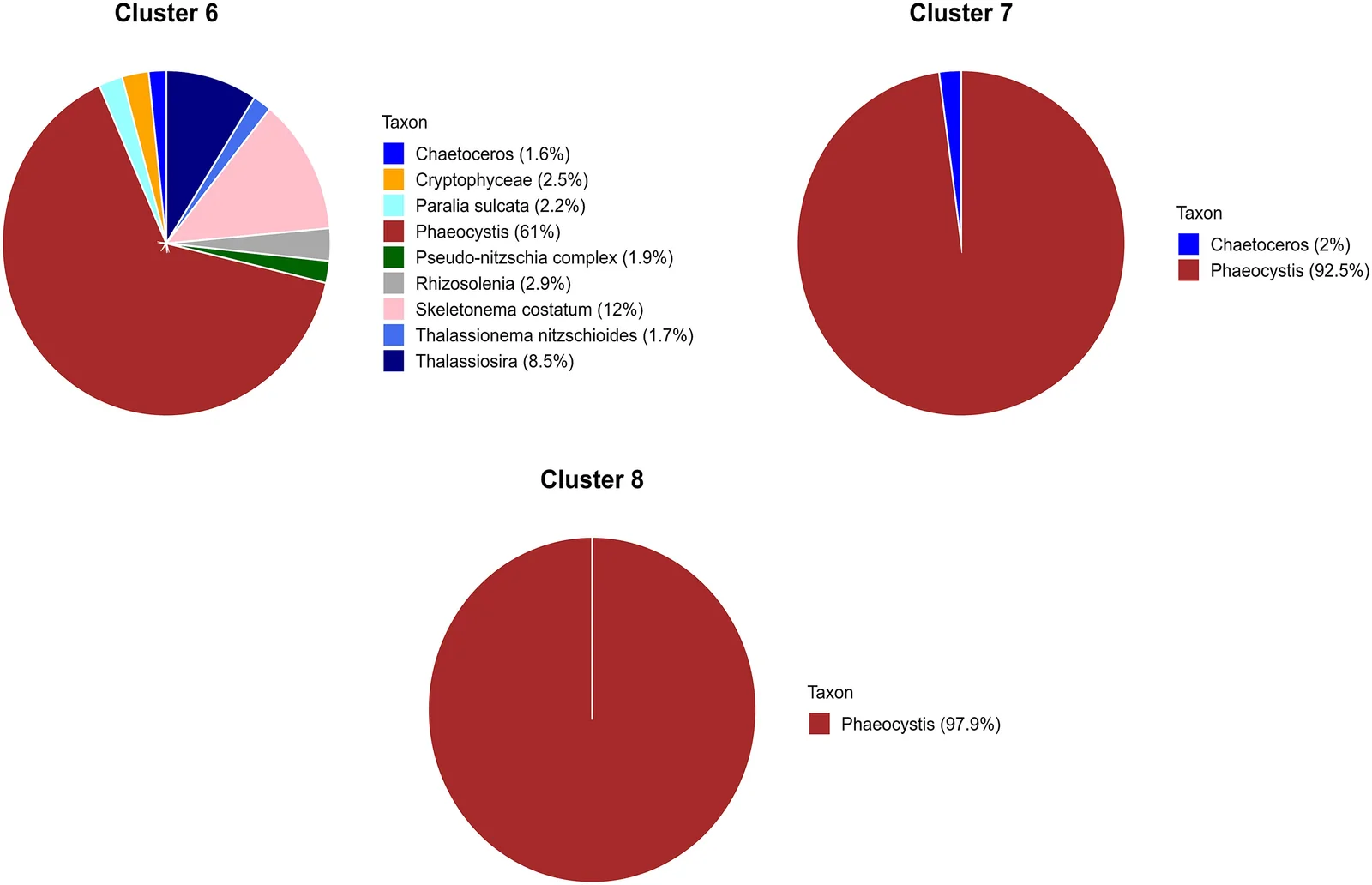
Relative abundance of dominant phytoplankton taxa (OTU) in clusters 6, 7 and 8. The cumulative abundance of the taxa represented in each of these clusters accounts for > 94% of the total abundance.

#### Transition state (part of spring and autumn period)

A transition state (Cluster 2) was identified between spring and summer on the one hand, and between summer and winter period on the other, representing periods of increasing nutrient availability following periods of low nutrient stock. This state extended from April to November (Fig. 1c), and was characterized by nutrient concentration higher than those observed during the second phase of the bloom (cluster 3), but lower than those of the winter period (cluster 5). Mean concentrations of nitrate + nitrite, phosphate and silicate were approximately 19, 0.9, and 5.6 μmol.L^−1^, respectively (Fig. 4). This transition state (Cluster 2) also supported a diverse phytoplankton assemblage, including *Pseudo-nitzschia* complex (18.6%), *Rhizosolenia* (15.6%), *Chaetoceros* (9.2%), *Cryptophyceae* (7.1%), and *Leptocylindrus* (3.6%) (Fig. 5).

#### Summer period

The summer period (Cluster 1) was marked by the highest water temperature (median = 18.2°C) and high PAR (mean = 432 μE.m^−2^.s^−1^), while exhibiting low biomass (fluorescence median = 0.76 FFU). It was characterized by low levels of nitrate + nitrite and silicate, with an approximate mean of 8 and 4 μmol.L^−1^, respectively (Fig. 4).

The most dominant phytoplankton taxon during the summer period was *Chaetoceros* (57.3%). These small species can be present from April or May to October. Other taxa such as *Cryptophyceae* (12.6%), and summer diatoms such as *Rhizosolenia* (6.4%), and *Guinardia* (4.6%) and *Gymnodiniales* (1.5%) can also be found in this period (Fig. 5).

#### Rare and extreme events

A rare event (Cluster 4), which occurred during a short period in 2008 and 2009, was identified. This event was so rare that it was not possible to link phytoplankton taxa to it. It was marked by high median of phosphate (0.94 μmoL L^−1^) and light (687.76 μE.m^−2^.s^−1^) (Fig. 4).

Moreover, an extreme event (Cluster 6) was identified, marked by unusually high nutrient concentrations, particularly nitrate + nitrite (approximate mean = 43 μmol.L^−1^), together with low salinity (approximate mean = 33 PSU; minimum = 26 PSU) (Fig. 4). Although both Cluster 5 and Cluster 6 were characterized by high nutrient concentrations and low salinity, Cluster 6 was classified as an “extreme” event because of its notably elevated nitrate-to-phosphate (N:P) ratio. Cluster 5 (winter period) exhibited an NP ratio of 37.6, whereas Cluster 6 showed a substantially higher ratio of 67.82, indicating atypical nutrient enrichment conditions beyond those of a normal winter period (Fig. 7). The phytoplankton assemblage associated with this state included *P. globosa (61%), S. costatum (12%), Thalassiosira (8.5%),* and *Chaetoceros (1.6%)* (Fig. 6).

**Fig. 7:**
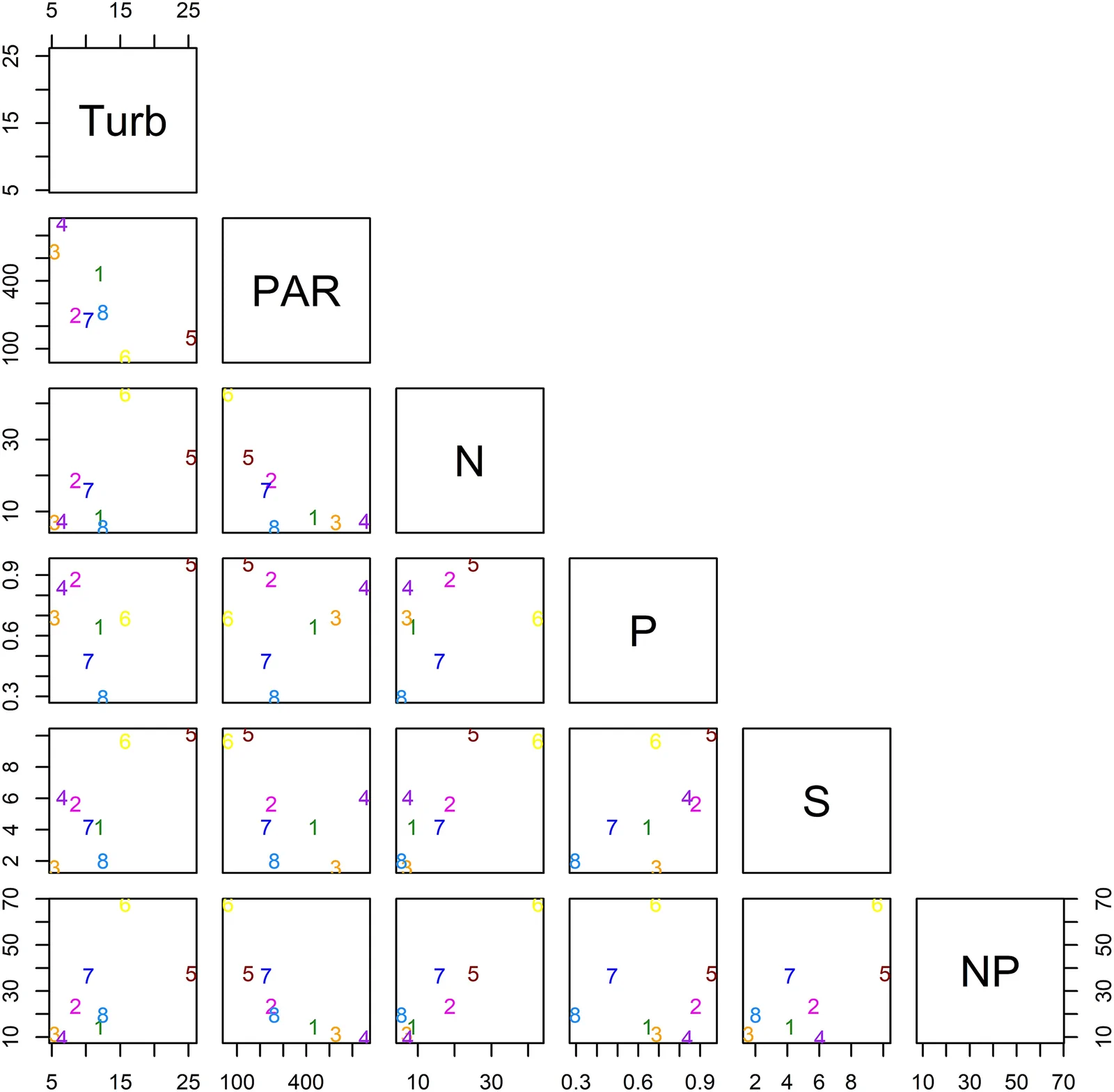
Margalef-based visualization diagram showing the environmental states (clusters) identified by their numbers in the eastern English Channel and the phytoplankton dynamics. The quantitative information for six variables, including turbidity (NTU), PAR (μE m^−2^ s^−1^), nitrate + nitrite (μmol L^−1^), phosphate (μmol L^−1^), silicate (μmol L^−1^), and NP ratio, can be retrieved for each environmental state.

### The Margalef-based visualization diagram

The Margalef-based visualization diagram shown in Fig. 7 provides a quantitative representation of the environmental conditions associated with each environmental state (cluster) and links each phytoplankton community to its corresponding environmental state. This diagram presents the barycenter of each cluster for six variables: turbidity (Turb), Photosynthetically Active Radiation (PAR), nitrate + nitrite (N), phosphate (P), silicate (S), and nitrate-to-phosphate ratio (NP). For example, the winter period (cluster 5) which included *S. costatum, P. sulcata, Rhaphoneis-Delphineis, T. nitzschioides, Thalassiosira,* and *Cryptophyceae* was characterized by low PAR (approximately 149.51 μE m^−2^ s^−1^), and high turbidity (approximately 25.39 NTU), as well as high nutrient concentrations. Nitrate + nitrite, phosphate, and silicate reached 25.08, 0.95, and 10.11 μmol L^−1^, respectively (Fig. 7), with an N:P ratio of approximately 37.6 (Fig. 8).

**Fig. 8:**
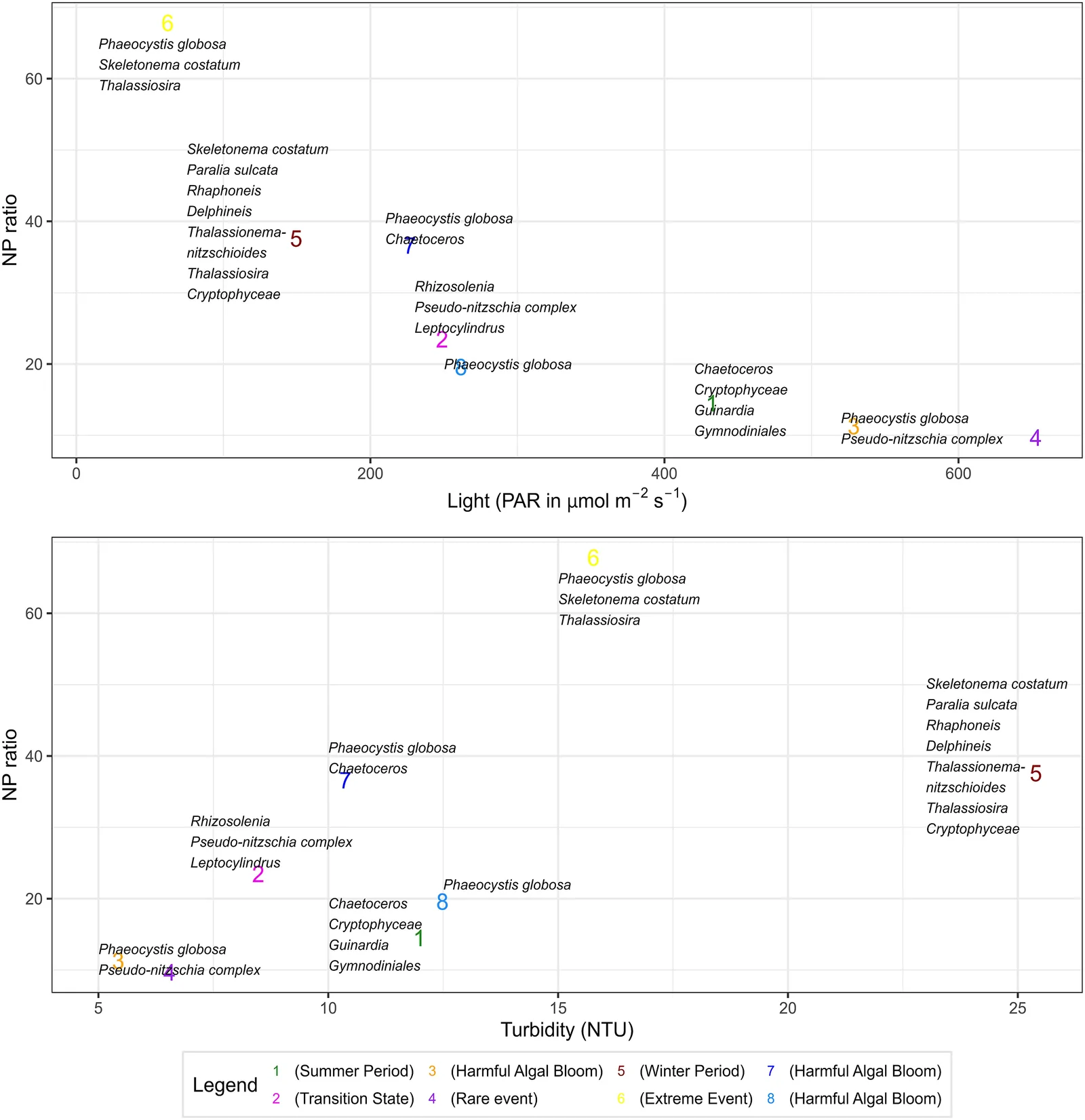
Projection of the main phytoplankton taxa along environmental gradients (NP ratio, light and turbidity) as identified by the Margalef-based visualization diagram.

At the end of the winter period, the increase in light PAR which reached 226.77 μE.m^−2^.s^−1^ initiated the spring bloom (Cluster 7) with *P. globosa* and *Chaetoceros*. This bloom was present at almost the same NP ratio as winter (36.61), but lower silicate (4.16 μmol L^−1^), phosphate (0.47 μmol L^−1^) and turbidity (10.37 NTU). When the spring bloom was associated with exceptionally high fluorescence, as observed in Cluster 8, it was characterized by the exclusive presence of *P. globosa* and comparatively low nutrient concentrations. This state was accompanied by higher turbidity (12.48 NTU) and exhibited a lower nitrate-to-phosphate ratio (19.6), together with lower nitrate + nitrite (5.57 μmol L^−1^), phosphate (0.29 μmol L^−1^) and silicate (2.01 μmol L^−1^) (Fig. 7 and Fig. 8). Next, the second phase of the harmful algal bloom (Cluster 3), dominated by *P. globosa* and *Pseudo-nitzschia* complex could be observed at higher light (528.76 μE.m^−2^.s^−1^), where nutrients were further consumed and NP ratio became very low (11.23), as indicated in Fig. 8.

A transition state (Cluster 2) follows the end of the bloom where diatoms including *Rhizosolenia*, *Pseudo-nitzschia* and *Leptocylindrus* predominate. During this state, nutrients regenerate as demonstrated by the increased NP ratio (23.47), and turbidity (8.48 NTU) as indicated in Fig. 8. This transition state was followed by the summer period (Cluster 1), during which *Chaetoceros, Cryptophyceae, Guinardia* and dinoflagellates (*Gymnodiniales*) could be observed, along with lower nutrients, a lower NP ratio (14.5), high light (432.21 μE.m^−2^.s^−1^) and low turbidity as indicated in Fig. 8.

In addition, an extreme event (Cluster 6) with a very high NP ratio of 67.82 at low light (62.10 μE.m^−2^.s^−1^) allowed the development of *P. globosa, S. costatum* and *Thalassiosira*. However, Cluster 4 (rare event) was marked by high phosphate, low NP ratio and light, as shown in Fig. 7, but no phytoplankton were identified.

The same characteristics can be derived for each environmental state. It is essential to highlight that the numerical values shown here correspond to the period 2005 to 2009 and may change in other years. From this Margalef-based visualization diagram, the phytoplankton dynamics in the eastern English Channel can be summarized, as shown in the revised Mandala (Fig. 9). It is evident that diatoms exist and thrive all year round, and not solely during periods of high turbulence and high nutrient concentration. This diagram can be used to derive the general pattern of phytoplankton seasonal succession in the eastern English Channel.

**Fig. 9:**
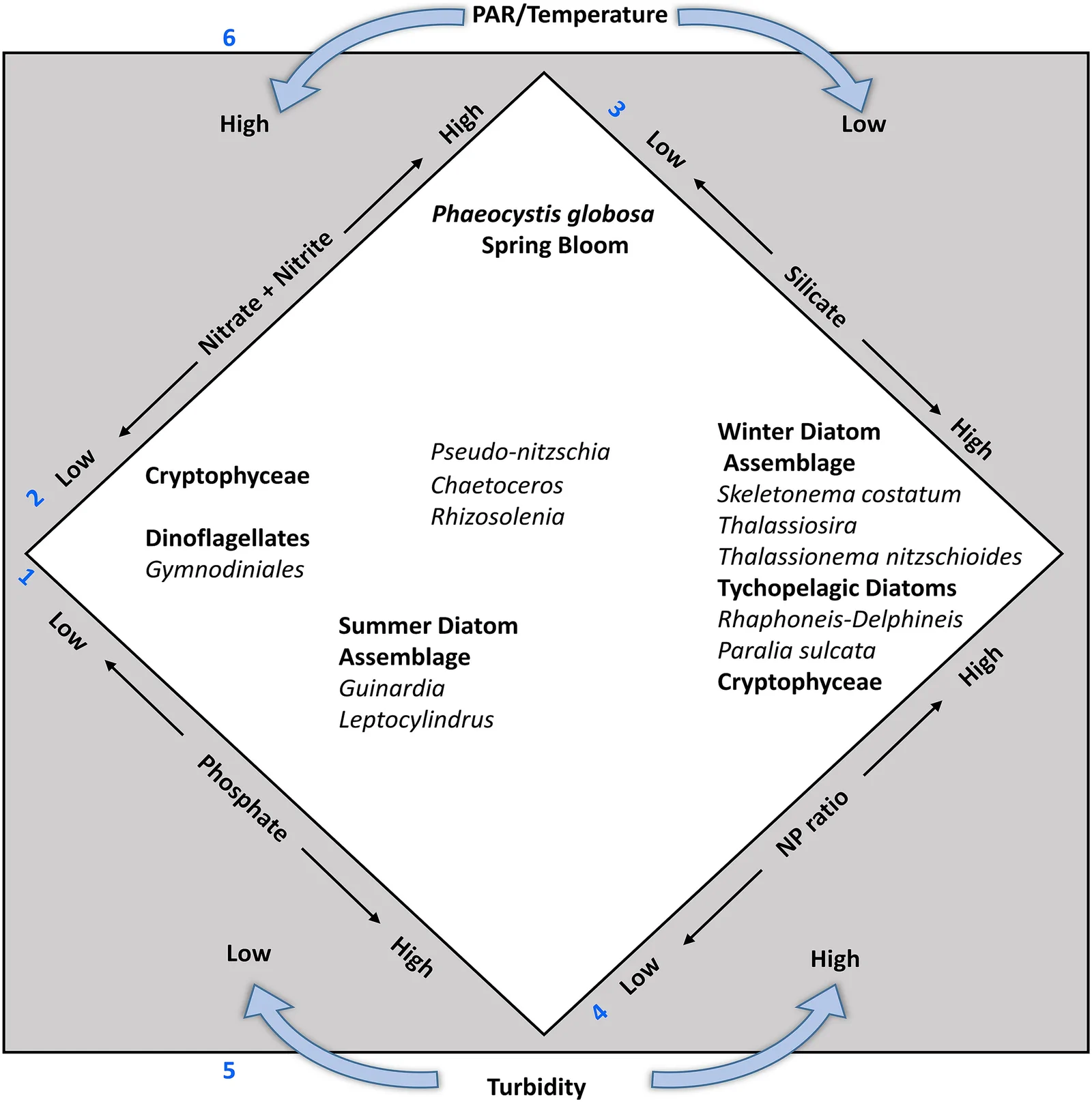
The revised mandala illustrating phytoplankton dynamics in the highly turbulent eastern English Channel, showing the relative low, intermediate and high values of turbidity, PAR, and nutrients (N: Nitrate + nitrite, P: Phosphate, S: Silicate, and NP ratio: Nitrate-to-phosphate ratio). The numbers represent the axes of the environmental gradients: (1) phosphate; (2) nitrate + nitrite; (3) silicate; (4) NP ratio; (5) turbidity; and (6) PAR and temperature. [modified from harmful algae, 55, Patricia M. Glibert, Margalef revisited: A new phytoplankton mandala incorporating twelve dimensions, including nutritional physiology, 25–30, copyright (2016), with permission from Elsevier].

The revised Mandala was subsequently tested using a recent dataset from the 2021 REPHY-SRN monitoring program. The results presented in Figs S1 and S2 show that the main seasonal succession patterns remain broadly consistent with those described by this Mandala. This confirms the robustness of the proposed Mandala under current environmental conditions.

To elaborate, interannual variability and climate-driven changes may influence phytoplankton community structure in the eastern English Channel, but the main ecological characteristics of the ecosystem remain comparable. The region is still characterized as a temperate, highly turbulent coastal ecosystem with turbid, nutrient-rich winter conditions, followed by spring blooms of *P. globosa*, transitional periods, and summer assemblages dominated by diatoms. More specifically, the winter period is still highly turbid, nutrient-rich and dominated by *Thalassiosira*, *T. nitzschioides*, *P. sulcata* and *Rhaphoneis-Delphineis*. This period is followed by spring blooms of *P. globosa* and *Pseudo-nitzschia* complex, which occur when silicate and phosphate become low while nitrate + nitrite remains high. Then, a transition period followed by a nutrient-poor summer period is defined where other diatoms such as *Rhizosolenia*, *Chaetoceros*, *Guinardia* and *Leptocylindrus* are observed at high temperature*. Cryptophyceae* are still observed in winter, transition and summer periods, while dinoflagellates are observed at low nutrient concentration during summer (Fig. S1 and S2). Consequently, no major shift or persistent transition toward dominance by other harmful taxa has been observed in the study area.

## DISCUSSION

Previous studies have identified the environmental states, phytoplankton dynamics and succession in the eastern English Channel using low-temporal resolution data (Schapira *et al.,* 2008; Lefebvre *et al.,* 2011; Hernández-Fariñas *et al.,* 2014; Skouroliakou *et al.,* 2024). Consequently, the interpretation of these states was mainly seasonally driven. It is necessary to shift towards high temporal resolution data to enhance understanding of environmental states and phytoplankton dynamics, given the complex benthic and pelagic interactions involved in eutrophication (Claussen *et al.,* 2009) and the multiple processes involved in phytoplankton dynamics at the spatial and temporal scale (Dickey and Bidigare, 2005). This study proposes a new numerical approach, which integrates high temporal resolution dataset from an in-situ instrumented station (MAREL Carnot) with low temporal resolution phytoplankton abundance data from the French monitoring program (REPHY-SRN). Besides, an unsupervised clustering approach was performed to identify distinct environmental states. This combination enhances knowledge of phytoplankton dynamics and environmental states, thus allowing the investigation of the seasonal variability and composition of phytoplankton in the well-mixed waters of the eastern English Channel, and the exploration of the abiotic environmental variables linked to their succession. A Margalef-based visualization diagram of this succession is consequently proposed.

To demonstrate, the novel approach proposed in this study permits the identification of rare, extreme and recurrent events linked to phytoplankton dynamics in the eastern English Channel. The extreme event (cluster 6) identified with an NP ratio of 67.82 deviating from the normal Redfield ratio (16), is likely a response to impulsive nutrients influx, which can be caused by floods. Moreover, the rare event (cluster 4) linked to phosphate resuspension in the region might be caused by sediment disruption, storms, or dredging activities. The exact determination of these states, however, requires complementary data that are not available/accessible at this time together with further analysis and knowledge.

Furthermore, the approach presented in this research highlights that diatoms do not respond homogenously to nutrient concentrations and underscores the ecological plasticity of diatoms, as well as that of *P. globosa*. For example, the winter period (Cluster 5) was dominated by several diatoms, including tychoplankton diatoms (e.g. *P. sulcata, Rhaphoneis,* and *Delphineis*). These bentho-pelagic diatoms appear in the water column after sediment resuspension due to intense water mixing mainly caused by wind-driven turbulence and strong tidal mixing. Other diatoms such as *S. costatum*, *Thalassiosira,* and *T. nitzschioides* were also characteristic of the winter period, consistent with Grattepanche *et al.* (2011a, 2011b) and Hernández-Fariñas *et al.* (2014). The existence of diatoms in winter coincided with high nutrient concentration, including silicate, which is needed for their silica shells. Despite the high nutrient concentrations, phytoplankton biomass remained low, consistent with the low light and temperature characteristics of the winter season, which are associated with low phytoplankton growth (Skouroliakou *et al.,* 2024).

Following the winter season, the decrease in silicate and phosphate together with the abundance of nitrate + nitrite, and increase in light (PAR) promoted the multiplication of *P. globosa* and their colony formation, highlighting the onset of spring blooms (Clusters 7, 8 and 3), consistent with Schapira *et al.* (2008), Lefebvre *et al.* (2011), Karasiewicz *et al.* (2018), and Breton *et al.* (2022a). *P. globosa* appears to form spring blooms along with other diatoms including the pennate diatom *Pseudo-nitzschia* complex which may release toxins in water, thereby rendering the bloom potentially toxic (Cluster 3). The abundance of *Pseudo-nitzschia* complex during *P. globosa* blooms has already been observed in previous studies (Sazhin *et al.,* 2007; Robache *et al.,* 2025) as an epibiotic commensal symbiotic relationship. In this study, domoic acid concentration was not available, however, Delegrange *et al.* (2018) reported toxic blooms of *Pseudo-nitzschia* in the region. Therefore, the future availability of domoic acid concentration data would allow a better distinction of harmful non-toxic and toxic algal blooms in the eastern English Channel.

During the spring bloom, nutrients were rapidly consumed, resulting in low concentrations (Clusters 8 and 3). *P. globosa* remained present and bloomed during periods of low phosphate availability (Cluster 8). This species was shown to express alkaline phosphatase activity, which may support its persistence during phosphate limitation (Ghyoot *et al.,* 2015).

Following the bloom, a transition state was identified, which was mainly observed between the spring bloom and summer, and then also between summer and winter. This state was characterized by increased nitrate + nitrite and silicate, consistent with a phase of post-bloom nutrient replenishment. The main phytoplankton assemblage in this state consisted of *Rhizosolenia*, the pennate diatom *Pseudo-nitzschia*, along with other *Protictista*. Then, under summer conditions characterized by high PAR, temperature, and low turbidity, diatoms again became dominant (Cluster 1), including the colony-forming *Chaetoceros*, and the centric diatoms *Leptocylindrus* and *Guinardia*. The observed assemblage is consistent with previous studies describing the seasonal occurrence of these taxa (Schapira *et al.,* 2008; Grattepanche *et al.,* 2011a; Grattepanche *et al.,* 2011b; Breton et al., 2022b; Skouroliakou *et al.,* 2024; Hubert *et al.,* 2025a), where they have been reported as characteristic of summer conditions in this temperate ecosystem, and are typically associated with higher temperature, light, and relatively lower silicate.

Other phytoplankton groups also contributed to the seasonal succession but exhibited distinct patterns of occurrence. In contrast to diatoms, dinoflagellates were limited to *Karlodinium* and *Gymnodiniales* and occurred at low abundance in the eastern English Channel, consistent with previous studies (Schapira *et al.,* 2008; Masquelier *et al.,* 2011; Hubert *et al.,* 2025a). *Cryptophyceae* were mainly observed during winter, as reported by Bonato *et al.* (2016), and during the transition state following the spring bloom and summer, consistent with previous observations (Bonato *et al.,* 2016; Robache *et al.,* 2025; Hubert *et al.,* 2025a). This seasonal distribution is also consistent with previous studies describing *Cryptophyceae* as a taxon capable of tolerating low light conditions, while also exhibiting mechanisms to cope with high light (Richardson, 2022, and references therein).

These results further demonstrate that phytoplankton communities in the highly turbulent waters of the eastern English Channel are dominated by diatoms and spring blooms of *P. globosa*. Specifically, these findings highlight distinct diatom assemblages associated with different environmental states. Based on these observations, a revised Mandala is proposed in this study. Compared with the Mandalas presented by Margalef (1978) and Glibert (2016), the Mandala presented in this manuscript separates diatoms along environmental gradients rather than separating diatoms from dinoflagellates.

The Mandala shown in Fig. 9 offers an overview of phytoplankton dynamics and succession in the eastern English Channel. Together with the phytoplankton succession presented in Fig. 8, it enables the identification of the drivers associated with this succession. Phytoplankton and trends can be read across more than two gradients simultaneously. Most importantly, the quantitative measurements provided by this approach could favour predictions of potential phytoplankton responses to climate change. Under increased nutrient availability, for example, one can predict the short-term future assemblage of phytoplankton based on the available information, which, in turn, can allow the development of future scenarios considering climate change. To demonstrate, Breton *et al.* (2022a) showed that climate change-driven decrease in nitrate may threaten diatom communities in the eastern English Channel. In our Mandala, *S. costatum*, *Chaetoceros, Rhizosolenia* were observed at higher nitrate levels, while *Leptocylindrus* and *Guinardia* were observed at lower nitrate concentrations. These results provide insight into how phytoplankton assemblages may respond to changing nutrient conditions linked to climate change and anthropogenic pressures. Consequently, a decrease in nitrate, for example, may favor *Leptocylindrus* and *Guinardia* over *S. costatum*, *Chaetoceros*, and *Rhizosolenia*.

While a Mandala presents a simplified model of phytoplankton dynamics, and no Mandala can include every detail, the nutrient axes here are differentiated into 4 gradients: silicate, nitrate +nitrite, phosphate and NP ratio. These axes are not representative of total nutrient loads as per Margalef (1978), nor do they include forms of nutrients such as ${NH}_4^{+}$ versus ${NO}_3^{-}$, or organic versus inorganic as per Glibert (2016).

In the Mandala presented in this research, diatoms are associated with high to intermediate silicate and phosphate concentration (Fig. 9). This distribution is consistent with their nutritional physiology as dissolved silicate is needed for silica shells (siliceous exoskeleton or frustules) (Martin-Jézéquel *et al.,* 2000), while phosphate is required by ribosomes to maintain high growth (Glibert, 2016).

Likewise, diatom community composition varied along the nitrate + nitrite gradient (Fig. 9). Diatoms are known to store nitrate intracellularly at concentrations that may exceed ambient levels (Stief *et al.,* 2022). Moreover, Stolte and Riegman (1995) indicated that phytoplankton cell size can influence nitrate uptake and storage, with larger diatoms capable of accumulating larger intracellular pools than smaller diatoms. This suggests that the diatom distribution along the nitrate + nitrite gradient may reflect differences in cell size related to nutrient acquisition and storage strategies. In contrast, dinoflagellates are placed at low nutrient concentrations, as they are mixotrophs.

Beyond nutrient availability, the revised Mandala places phytoplankton taxa across gradients of turbidity, light (PAR) and temperature, which may provide insight about photoacclimation and efficient light use by different taxa. Nonetheless, further variables/studies are needed to enhance knowledge of adaptation and photoacclimation. Light conditions, defined by PAR, varied across the sampling season and coincided with changes in phytoplankton composition (Fig. 9). Increased light (PAR) can induce oxidative stress, leading to enhanced antioxidant production in phytoplankton (Allen and Polimene, 2011). This suggests that the revised Mandala may reflect patterns consistent with phytoplankton antioxidant production in response to light variability. Notably, *P. globosa* is an exception in this regard, as it is known for dimethyl sulfide production during spring blooms (Riegman and Van Boekel, 1996). The revised Mandala also distinguishes taxa according to motility, with all cells being immotile except dinoflagellates, *Cryptophyceae* and solitary cells of *P. globosa*.

Interestingly, our numerical approach allowed the identification of blooms of *P. globosa* in small proportions in December and February. These blooms result from the ability of the genus *Phaeocystis* to develop at low light intensities (Moisan and Mitchell, 1999). Although previous research by Bonato *et al.* (2016) highlighted the ability of *Phaeocystis sp*.to grow under low light, our findings further highlight the need to adapt monitoring strategies. This means that in-situ low-frequency monitoring networks, such as REPHY-SRN, should consider increasing sampling during low-light months to better track and understand phytoplankton dynamics, and hence better evaluate environmental states and issue recommendations for coastal ecosystems management, in alignment with Caillault and Lefebvre (2017).

The main limitation of M-SC is its requirement for a complete dataset to capture ecosystem dynamics. Although the data completion approach proposed in this study (DTWBI) was necessary to address missing values, it may lead to an overestimation of recurrent events relative to rare ones. These considerations should be taken into account when interpreting the clusters, particularly given the inherent complexity of marine ecosystems.

Moreover, the monthly to fortnightly phytoplankton abundance dataset may limit the ability to link phytoplankton taxa to rare events. In addition, the phytoplankton dataset is restricted to microphytoplankton, preventing the assessment of nano- and pico-phytoplankton and limiting conclusions regarding potential shifts toward smaller size classes. This is particularly relevant given that Hubert *et al.* (2025b) highlighted a shift towards smaller phytoplankton in the eastern English Channel. The implementation of flow cytometry could help overcome these limitations by providing higher-frequency observations and a broader characterization of phytoplankton classes and functional groups. The long-term acquisition of such data from instrumented stations such as MAREL Carnot in the future could constitute an interesting improvement for the numerical analysis and ecological interpretation proposed in this research. Indeed, a recent study by Robache *et al.* (2025) has already taken steps in this direction, with automated flow cytometry installed on MAREL Carnot as a proof of concept, enabling a detailed characterization of phytoplankton functional group succession in the eastern English Channel. The application of the numerical approach proposed in this study to such a dataset would allow for a more detailed description and improved understanding of the highly turbulent English Channel, its environmental states, and its phytoplankton dynamics.

Ultimately, no Mandala can represent everything; otherwise, it would be reality rather than a Mandala. The Mandala presented in this study lacks zooplankton grazing due to the absence of relevant data. Future studies can further incorporate this type of data to enhance studies in the eastern English Channel regarding top-down mechanisms of phytoplankton seasonal succession.

Lastly, this study further highlights the need to consider longer time series to capture more extreme, rare and recurrent environmental states. This would improve the capacity to assess the effects of changes in local pressures associated with environmental management and climate change, as well as to detect potential regime shifts in the studied ecosystem.

## CONCLUSION

This study investigates phytoplankton seasonal variability, succession and composition in the well-mixed coastal waters of the eastern English Channel, and explores the abiotic environmental variables linked to their succession. A new numerical approach is proposed to combine a high-frequency hydrological and physicochemical dataset (MAREL Carnot) with a low-frequency phytoplankton abundance dataset (SRN-REPHY). This approach represents a methodological shift from low-frequency analyses relying on traditional statistical approaches to a novel integration of high-frequency data with low-frequency datasets, using machine learning to extract deeper insights from complex ecological patterns.

The M-SC allows the identification of eight different environmental states associated with phytoplankton dynamics in the eastern English Channel including rare, extreme and recurrent events. M-SC therefore enables the differentiation of various phytoplankton assemblages across seasons at high temporal resolution (20 minutes). It also allows the distinction between harmful non-toxic blooms of *P. globosa* and potentially toxic blooms where *Pseudo-nitzschia spp*. are present.

The Margalef-based visualization diagram links environmental states to six environmental variables: turbidity, light (PAR), nitrate + nitrite, phosphate, silicate, and nitrate to phosphate ratio (NP ratio). By linking phytoplankton taxa to environmental states, the diagram provides a framework for relating phytoplankton to environmental variables, and improves understanding of phytoplankton dynamics and succession in the eastern English Channel. This framework could possibly be extended to other highly turbulent coastal areas. Moreover, this study demonstrates how different environmental drivers, such as nutrient availability, light, and turbidity, shape phytoplankton community structure and bloom dynamics over time. Consequently, this approach may enables performing some predictions of phytoplankton assemblages in the context of climate change and anthropogenic pressure. Further research is needed to include nano- and pico- phytoplankton as well as zooplankton to improve our comprehension of phytoplankton dynamics.

Future studies of phytoplankton dynamics and environmental states may benefit from such an integrated observational approach. Integrating data from the most pertinent monitoring systems (buoys, in-situ, flow cytometry, satellite and modeling) with optimal numerical methodologies suited to spatial and temporal data may facilitate the extraction of relevant information and provide a more comprehensive understanding of phytoplankton dynamics and environmental states.

## Supplementary Material

Accepted_Changes_Supplementary_Materials_fbag086

## Data Availability

The data underlying this study are available from the corresponding data repositories. The MAREL Carnot dataset is freely accessible through SEANOE (https://doi.org/10.17882/39754). The REPHY-SRN dataset was obtained from Quadrige2 and is publicly and freely accessible through SEANOE (https://doi.org/10.17882/47248).

## Notes

Corresponding Editor: Lisa Campbell
