# Supplementary material for: Phytoplankton seasonal succession in response to environmental drivers in the eastern English Channel: a revised Margalef Mandala for a highly turbulent region
Source: J Plankton Res. 2026 Nov 12;48(6):fbag086. doi: 10.1093/plankt/fbag086 (PMC13755355; doi:10.1093/plankt/fbag086)
Supplement: Supplementary file 1 [file accepted_changes_supplementary_materials_fbag086.docx]

**Phytoplankton Seasonal Succession in Response to Environmental Drivers in the Eastern English Channel: A Revised Margalef Mandala for a Highly Turbulent Region**

*Raed HALAWI GHOSN^1^*, *Kelly GRASSI^1,2,^***‡**, *Emilie POISSON-CAILLAULT^2^*, *Alain LEFEBVRE^1^*

^1^Ifremer, Unité COAST, Laboratoire Environnement et Ressources, 62200 Boulogne-sur-Mer, France

^2^LISIC, UR 4491 ULCO-Université du Littoral Côte d’Opale, 62228 Calais, France

**‡** Present address: CLS Group (Collecte Localisation Satellites), 31000 Toulouse, France

**Corresponding authors:** Raed HALAWI GHOSN and Alain LEFEBVRE

**Contact details:**

Raed HALAWI GHOSN: [raed.halawi.ghosn@ifremer.fr](mailto:raed.halawi.ghosn@ifremer.fr); ORCID: <https://orcid.org/0009-0008-4532-4726>

Kelly GRASSI: [kgrassi@groupcls.com](mailto:kgrassi@groupcls.com) ; ORCID: <https://orcid.org/0000-0003-2104-3686>

Emilie POISSON-CAILLAULT: [emilie.caillault@univ-littoral.fr](mailto:emilie.caillault@univ-littoral.fr) ; ORCID: <https://orcid.org/0000-0001-6564-8762>

Alain Lefebvre: [Alain.Lefebvre@ifremer.fr](mailto:Alain.Lefebvre@ifremer.fr); ORCID : <https://orcid.org/0000-0003-4794-874X>

**Supplementary Materials**

Figure S 1 Phytoplankton seasonal succession observed from REPHY-SRN dataset for the year 2021. The abundance of operational taxonomic units is provided in (Cell L-1)

Figure S 2 Seasonal variability of environmental variables including nutrients (nitrate + nitrite (µmol L^-1^), phosphate (µmol L^-1^), silicate (µmol L^-1^), water temperature (°C) and turbidity (FNU)
